# Type 1A Endoleak after TEVAR in the Aortic Arch: A Review of the Literature

**DOI:** 10.3390/jpm12081279

**Published:** 2022-08-04

**Authors:** Lucia Scurto, Nicolò Peluso, Federico Pascucci, Simona Sica, Francesca De Nigris, Marco Filipponi, Fabrizio Minelli, Tommaso Donati, Giovanni Tinelli, Yamume Tshomba

**Affiliations:** 1 Unit of Vascular Surgery, Fondazione Policlinico Universitario A. Gemelli I.R.C.C.S., Università Cattolica del Sacro Cuore, 00168 Roma, Italy; 2 Unit of Vascular Surgery, Fondazione Policlinico Universitario A. Gemelli I.R.C.C.S., 00168 Roma, Italy; 3 ASL Roma 2, 00168 Roma, Italy

**Keywords:** aortic arch, TEVAR, endoleak, chimney, fenestrated graft, branched graft, in situ fenestration

## Abstract

Aortic arch repair is a challenging intervention. Open surgical repair is still considered the gold standard, but in high-risk patients, it is not always a reasonable option, making endovascular approaches an enticing, when not the only available, alternative for treatment. The strategies more commonly adopted are surgical supra-aortic trunk (SAT) rerouting followed by deployment of a standard thoracic endoprosthesis, chimney techniques, custom-made scalloped, fenestrated, and branched devices, and in situ or physician-modified fenestrations. If we excluded techniques involving SAT rerouting where the arch anatomy is surgically modified in order to make deployment in the aortic arch of a standard thoracic endoprosthesis possible, in the other techniques, one or more SATs are incorporated in the thoracic endoprosthesis. In these cases, no matter what solution is adopted, because of the morphology of the aorta at this level, achieving an ideal sealing is extremely difficult, and endovascular treatments of the arch are burdened by an increased risk of type IA endoleaks. PubMed, EMBASE, and Cochrane Library were searched. We identified 1277 records. After reading titles, abstracts, and full texts, we excluded 1231 records. Exclusion criteria were low-quality evidence, abstracts, case reports, conference presentations, reviews, editorials, and expert opinions. A total of 48 studies were included, for a total of 3114 patients. A type IA endoleak occurred in 248 patients (7.7%) with a mean incidence of 18.8% in chimney procedures, 4.8% and 3%, respectively, in fenestrated and branched devices, and 2.2% in in situ fenestration. We excluded from our analysis scalloped technology that is used when the target vessel originates from a healthy landing zone and represents a different anatomical setting. Type IA endoleaks are a concern with all types of endovascular aortic arch repair, and they can compromise the outcomes of the procedure. The rate of type IA endoleaks appears to be significantly higher in chimney procedures. In order to maximize sealing, whenever possible, endovascular repair of the arch should be achieved with custom-made fenestrated devices. However, chimney configurations are still a valuable solution particularly in the emergency setting, although in such a procedure, to guarantee accurate postoperative management and follow-up, an imaging protocol could be useful.

## 1. Introduction

Open surgical aortic arch replacement is one of the most challenging procedures in cardiovascular surgery due to both procedural criticalities, mainly due to the technical complexity and the challenges of cerebral protection, and the physiological impact such a burdensome surgery has especially on frail patients. Open surgical repair still remains the gold standard, yet, in high-risk patients, it is not always considered a reasonable option. With the development of endovascular techniques and devices for the treatment of aortic disease, the use of thoracic endovascular aortic repair (TEVAR) in highly selected cases has been expanded to the aortic arch. However, TEVAR for the treatment of aortic arch disease still represents a challenge due to the aortic arch morphology, the effluence of the supra-aortic trunks, the peculiar hemodynamics, and the stress during the cardiac and respiratory cycles [1]. Even in this complex environment, endovascular techniques can be a valuable option, in a selected group of patients after thoughtful and accurate preoperative planning of the strategy and the various steps of the procedure. Strategy, technique, landing zones, and stent graft choice are all crucial criteria that can dictate this procedure’s outcomes, particularly the occurrence of proximal endoleaks.

In this paper, we aim at describing how the occurrence of type IA endoleaks affects different techniques and which role it plays in the postoperative outcomes and the need for secondary procedures. We chose to focus only on type IA endoleaks due to the fact that the significance of differences among different techniques is not as noticeable in other types of endoleaks.

## 2. Materials and Methods

### 2.1. Study Search Strategy

This systematic review was conducted according to the guidelines of the Preferred Reporting Items for Systematic Reviews and Meta-Analyses (PRISMA) [2]. A literature search was performed by three independent authors (L.S., F.P., and N.P.) based on three databases (PubMed, EMBASE, and Cochrane Library) and was focused on the period between January 2012 and March 2022 to keep our selection as contemporary as possible. To achieve the maximum sensitivity of the search strategy, the terms “Endovascular aortic arch repair” or combinations were searched with some common expressions referring to such procedures resulting in the following string: (therapy/broad[filter]) AND ((aortic arch repair OR aortic arch endovascular repair) AND (aorta OR aortic) AND (endoleak) AND (branched OR fenestrated OR in situ fenestration OR chimney)).

The references of all articles and reviews on the topic and top hits from Google Scholar were also scrolled to identify other potentially relevant studies. To avoid overlap, the International Prospective Register of Systematic Reviews (PROSPERO), an open-access online database for systematic reviews, was inquired. PROSPERO is an open-access online database for systematic reviews, which allowed us to avoid topics already widely analyzed in the literature.

### 2.2. Selection Criteria

Studies investigating different strategies of aortic arch endovascular repair and its outcomes were deemed eligible. The titles and abstracts were screened, and only clinical studies published in peer-reviewed journals in the English language were considered. We excluded duplicates, studies with missing data, abstracts, case reports, conference presentations, reviews, editorials, and expert opinions. Article selection was independently performed by three authors (L.S., F.P., and N.P.); disagreements were resolved by discussion and consensus (the same approach was used for all tasks performed by more than one investigator).

### 2.3. Data Extraction and Criteria Appraisal

Data were extracted from article texts, tables, and figures and included the title, year of publication, study design, sample size, study population, patient characteristics, outcomes, findings, and conclusions. Three investigators (L.S., F.P., and N.P.) independently reviewed each article. Data from full-text articles were entered into an Excel spreadsheet with structured tables.

### 2.4. Study Selection

**Figure 1:**
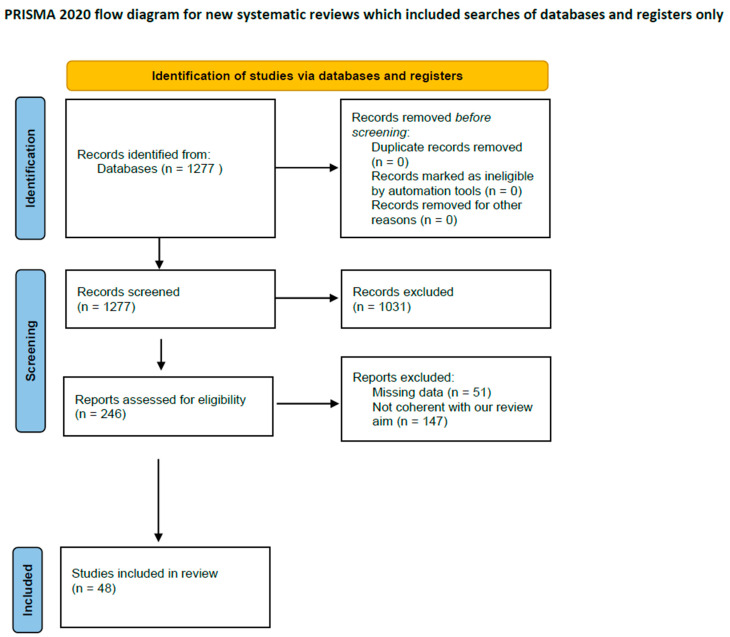
PRISMA flowchart.

The search generated n = 1277 articles; according to the previously described approach, after the reading of the title and abstract, n = 72 manuscripts were eligible for a comprehensive evaluation. After full-text reading, we ultimately selected n = 49 articles that satisfied the prespecified selection criteria; n = 23 manuscripts were conversely excluded because either the topic did not address the study question (n = 9) or because of missing data (n = 2) (Figure 1). Reference lists from the selected papers were also screened.

## 3. Results

Based on our research strategy, 248 type IA endoleaks were recorded in a total of 3114 patients (7.7%). All patients were matched for age, risk factors, and procedural factors.

Out of the 48 studies selected: 10 [3,4,5,6,7,8,9,10,11,12] described outcomes in chimney procedures, 13 [1,13,14,15,16,17,18,19,20,21,22,23,24] with the use of fenestrated devices, 9 [25,26,27,28,29,30,31,32,33] focused on in situ fenestration outcomes, 14 [34,35,36,37,38,39,40,41,42,43,44,45,46,47] on branched devices, one study compared fenestrated and branched devices [48], and one compared in situ fenestration and the chimney technique [49].

In the first group, out of 964 patients who underwent chimney procedures, 182 type IA endoleaks were identified (18.8%). In the second group, out of 575 patients who underwent procedures using fenestrated stent grafts, a type IA endoleak occurred in 28 cases (4.8%). In the third group, out of 528 patients on which TEVAR with in situ fenestration was performed, 12 type IA endoleaks occurred (2.2%). In the fourth group, out of 761 patients who underwent procedures with branched devices, 23 type IA endoleaks were described (3%). Our results are described in Table 1.

### 3.1. Chimney Technique

A total of 897 patients were analyzed, with a mean type IA endoleak incidence of 20.1% (from 7.4% to 40%).

Among all inquired studies, none of the experiences was free from type IA endoleaks. All reported endoleaks were type IA endoleaks related to the presence of gutters inherently linked to this kind of technique. The average incidence ranged from 7.4% (2 out of 27), as reported by Voskresensky [8] et al., to 40% (22 out of 55), as reported by Kanaoka et al. [6]. Other incidences described were 14.8% (8 patients out of 54 reported by Ahmad et al. [3]), Bosiers et al. [4] 10.5% (10 out of 95), Huang et al. [5] 16.3% (37 out of 226), Mangialardi et al. [7] 23% (6 out of 26), Wang et al. [9,10] 13 (3 out of 23) and 10.6% (13 out of 122), Zhao et al. [11] 32% (75 out of 234), and Zou [12] et al. 14.2% (5 out of 35). Most of them were described as intraoperative endoleaks.

### 3.2. Fenestrations

A total of 556 patients were analyzed, with a mean type IA endoleak incidence of 5% (from 0% to 21.4%).

Furuta et al. [1] reported a recent and large experience with the use of fenestrated stent grafts: among 205 aortic repairs, 7 type IA endoleaks occurred, with a 3.4% incidence. Fernandez-Alonso et al. [13] described 1 type IA endoleak among 14 patients, but it was not detectable at discharge. Canaud et al. [14,15] (with two patient samples, one of 35 patients and one of 17), Tsilimparis et al. [20] (44 patients), Tan et al. [19] (7 patients), Yap et al. [21] (5 repairs), Zhou et al. [22] (42 patients), and Zhu et al. [23] (10 patients) all reported the absence of endoleaks in their samples both at discharge and at follow-up. In a more recent experience, Zhu et al. [24] described 58 fenestrated graft implants with 2 cases of type IA endoleaks, both linked to deployment failure. Chassin-Trubert et al. [16] described 1 case of type IA endoleak in a sample of 50 patients. A total of 3 type IA endoleaks were reported among 32 procedures by Iwakoshi et al. [17], while the highest endoleak incidence was reported by Kurimoto et al. [18] with an incidence of 32% (12 patients out of 37).

### 3.3. In Situ Fenestration

A total of 510 patients were analyzed, with a mean type IA endoleak incidence of 2.3% (from 0% to 4.7%).

Three studies (Hu et al. [26] reported a series of 107 patients, Katada et al. [27] 7 patients, and Wang et al. [32] 5 patients) reported no cases of periprocedural or long-term endoleaks. Likewise, Le Hoeurou et al. [29], Luo et al. [30], and Redlinger et al. [31] in their respective series described only type II and III endoleaks. Two more authors reported the occurrence of type IA endoleaks which ranged from 2% (Zhao et al. [33] with 4 out of 130 patients) to 5% (Li Chong et al. [25] with 7 out of 148 patients). Kopp et al. [28] instead described 1 type IA and 4 type III endoleaks out of 25 patients.

### 3.4. Branched Endografts

A total of 744 patients were analyzed, with a mean type IA endoleak incidence of 4.8% (from 0% to 18.1%).

Clough et al. [35], Ferrer et al. [38,39], and Dai et al. [37] did not report any type I endoleaks. Type IA endoleaks were described by Chen et al. [34] (1 in 122 patients), Czerny et al. [36] (1 in 43 patients), Kawajiri et al. [40] (2 in 11), Patel et al. [42] (4 in 22 patients), Tazaki et al. [43] (8 in 217 patients), Verscheure et al. [46] (2 in 70 patients), and Tsilimparis et al. [44,45] (with two experiences in which 2 out of 54 patients and 1 out of 20 patients), with an incidence ranging from 0.7 to 20%. A type 1B endoleak was described by Czerny et al. [47] (1 in 43 patients), Li et al. [41] (1 in 16), and Tazaky et al. [43] (1 in 217). Konstantinou et al. [48] in a comparative study among 19 fenestrated and 17 branched devices described a type IA endoleak in the latter group.

## 4. Discussion

Endovascular repair of the aortic arch is undoubtedly a complex and challenging procedure, and outcomes can be affected by a multitude of factors. One of the main concerns in this aortic zone is related to the need for SAT inclusion which parallels the challenges of visceral vessel inclusions in the thoracoabdominal aortic segment [50]. Looking at the literature, branched, fenestrated, in situ fenestration, and chimney configurations all represent valid options, with acceptable complication rates, despite which condition is being treated. According to data extracted in our literature analysis, regardless of the chosen device or technique, the technical success ranged between 97 and 100%.

In situ fenestration is an off-label procedure that allows customizing off-the-shelf devices, especially in emergency settings, using either sharp or energy-based techniques. In urgent cases, deploying a thoracic endograft within the aortic arch with the subsequent creation of in situ retrograde fenestrations allows supra-aortic trunk revascularization. It is a challenging technique that requires the availability of a wide range of materials and a skilled team and likely involves a learning curve. One of the main strengths of this solution is that it can be used in both elective and emergency settings. For urgent cases, it is worth reminding that customization of a stent graft for the treatment of the arch would require on average between 6 and 8 weeks allowing treatment only for elective cases. Endoleak rates in such procedures are infrequent, with the lowest incidence of such complications among all investigated techniques (2.3%). However, in situ fenestration in the arch is still burdened by a significant rate of complications (3.4%) [51], with the risk of aortobronchial or aortoesophageal fistulas.

In selected cases, fenestrated stent grafts can be a valid solution for the treatment of limited arch lesions when supra-aortic trunks originate from the diseased aorta (as opposite to scallop devices, where the SATs originate from a healthy vessel and therefore can be spared). This technique requires good apposition of the stent grafts to the aortic wall at the level of the gaps created in the fabric of the stent graft to preserve flow to the supra-aortic trunks; hence, in most circumstances, it is not indicated to treat circumferential dilatations of the arch. In patients with aortic arch disease and short proximal landing zones, the use of fenestrated stent grafts increases sealing length and enables accurate deployment while maintaining supra-aortic trunk patency [13]. Fenestrated stent grafts can be associated with stenting or not, and while some authors stated that no statistically significant difference in endoleak rates was described [16], others [17] strongly advised supra-aortic stenting due to the way in which the aortic arch curvature could affect device positioning, reporting a significant success rate and a 97% 3-year patency rate. With fenestrated stent grafts, the rationale for stenting is not so much to achieve better sealing but to correct and or avoid misaligning between fenestrations and the ostia of the target vessels. This can be achieved with both off-the-shelf devices and physician-modified ones, in order to adapt the device to the patient’s anatomy in cases in which a custom-made device is not a suitable option. Although it might be appealing to avoid the uncertainty of stent grafts, alignment stenting requires extra manipulation of the supra-aortic trunks which increases the risk of a stroke.

**Figure 2:**
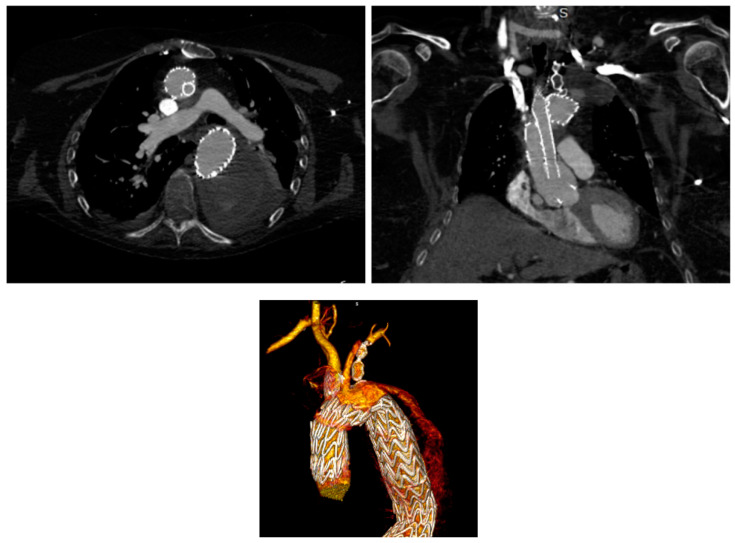
An example of chimney technique and its volume rendering, where we can observe the endoleak.

Branched devices (Figure 2) aim at achieving an anatomical reconstruction of the arch allowing treatment of extensive disease with complete exclusion of the aortic segment involved. Sealing within the supra-aortic trunks reduced the risk of endoleaks that might arise at the level of gutters and or fenestrations; however, it comes at the cost of multiplying the sealing zones, hence increasing the complexity and variables of the interventions. In the literature, type 1 endoleak rates associated with such procedures are lower than other techniques, yet, they are linked to other complications such as cerebrovascular events [51] or retrograde dissection [52]. Ferrer et al. reported their experience in which no endoleaks occurred but with a 25% cerebrovascular event rate and an 8.3% rate of retrograde dissection. Of note, no reintervention was necessary in their series. Fenestrated and branched devices aim at achieving direct sealing in the arch; however, the anatomical requirements differ. For how much branched solutions can be applied to widely different anatomical configurations, fenestrated stent grafts positioned across the arch, as stated previously, require wall apposition at the level of the fenestrations and would not accommodate lesions where this is not guaranteed at the level of the ostia of the supra-aortic trunks. As a consequence of the limitations and the complexity of accurate deployment of the devices at the level of the arch, a strict postoperative protocol, inclusive of an early CTA, should be adopted in all cases regardless of the technique in order to allow early identification and correction of technical issues that might be identified [40].

**Figure 3:**
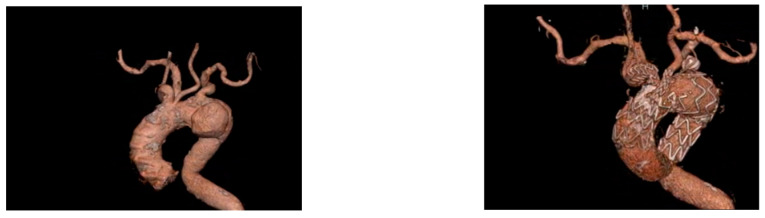
Before and after a total endovascular aortic arch repair using double-branched Terumo Aortic.

The chimney technique (Figure 3) is commonly used in both emergent and elective patients and has been widely described in the literature, with high success rates but still a significant risk of endoleaks, especially type 1A ones (20.1% in our analyzed studies). The choice of the ideal stent for the chimney extension is debatable; choosing the right stent in the arch has to take into account very specific factors which differ significantly from the technique employed in the abdominal aorta. Those factors include the radial force of the different thoracic stent grafts, the amount of oversizing of the stent graft, diameter, angles, and possible tortuosity of the sealing zones at the level of the target vessels, and challenges related to the access. Both balloon-mounted and self-expanding, (bare or covered) stents have been applied, but there is no consensus and only a little science about which one is the ideal stent [5]. Intuitively and as widely stressed by many authors in the literature, preoperative planning is key and should be thoroughly conducted. In particular, at least 10 mm of the healthy aortic neck should always be granted to ensure adequate sealing, while the main-graft–parallel-graft complex acts as a fixation zone to provide structural stability [7].

Gutter endoleaks seem to be the most predictable procedural failure, with incidence rates that could be as high as 44%. No difference among landing zones emerged, yet, zone 0 procedures were associated with a higher complication risk, due to the need for total debranching to preserve supra-aortic branches [11]. While, in other techniques, endoleaks appear especially from the 30-day follow-up on, in chimney procedures, almost all type I endoleaks can be detected intra or immediately postoperatively as early as during the procedure. As gutter endoleaks can resolve in the days and weeks following the implant, it is arguable that in the case of a chimney configuration, a different definition of technical failure might need to be adopted, and the focus should be on delayed more than early type I endoleaks.

## 5. Limitations

Due to the complexity of the topic, the relatively small numbers of patients, limited median follow-up, and the heterogeneity of available literature, our study had multiple limitations.

The main one consisted in lacking data. In particular, few studies reported the clinical outcomes and management of occurred endoleaks; hence, we could not discriminate whether or not they required treatment or caused ulterior complications (such as stent malfunctioning or aneurysmatic sac growth and aortic rupture). It has been stated that frequently such endoleaks tend to resolve themselves, yet, it was not possible from the published data to assess the reliability of such a statement. Furthermore, many papers reported short follow-ups; hence, the lack of long-term data prevents us from understanding the consequences of endoleaks in terms of reintervention and aortic-related events.

Furthermore, although the SVS Reporting Standards for endovascular aortic aneurysm repair (EVAR) consider the presence of a type I or III endoleak a technical failure, there are no specific reporting standards regarding the treatment of the arch. Authors often fail to distinguish the success rate of the execution of the technique and deployment of the grafts from the formal definition of technical success rate, which should also include the absence of type I A endoleaks. Moreover, it has to be acknowledged that chimney configurations should somehow fit in a category apart as gutters—with evidence of a proximal endoleak around the graft—have to be expected at the final angiogram in almost every procedure.

We tried to sort out whether or not the type of aortic arch pathology could affect endoleak incidence rates, but we did not find any statistical significance. We did not analyze how different complications may have different incidence rates though, since it was not our main goal. A bit more complex appears to be the influence of the proximal landing zone. Though it appears not to be linked itself to the type IA endoleak incidence (as previously stated), it is true that it can influence the choice of technique and create a bias that is difficult to overcome. Still, having chosen to focus more on how this kind of complication affects postoperative management in such patients than how to lower its rate, we decided to go through with our analysis.

Moreover, in several papers, the choice of the devices was not specified. Due to the high variability of marketed devices, it was not possible to evaluate the incidence of endoleaks when using different devices.

## 6. Conclusions

With few exceptions, open surgical repair remains the gold standard for the treatment of conditions affecting the aortic arch. In frail patients deemed too high risk for open repair as well as in emergency settings, total endovascular repair, when local expertise and appropriate set-up are available, is a reasonable solution providing acceptable outcomes. Type IA endoleaks are a concern with all types of endovascular aortic repair as they can compromise the outcomes of the procedure [35]. Available data, though, lack uniformity and comparability. Not surprisingly, due to specific issues with the gutters, rates of type I endoleaks appear to be significantly higher in chimney procedures. As they are particularly common after endovascular repair of the aortic arch, for the purpose of early identification and, when possible, treatment of type IA endoleaks, a rigorous postoperative imaging follow-up should be guaranteed for all patients. As much as there is a clear need for standardization of protocols and materials, patients undergoing total endovascular repair of the arch will always need highly tailored interventions which should ideally be performed in high-volume centers with an appropriate set-up and expertise.

Furthermore, in order to guarantee appropriate postoperative management and follow-up, tailored protocols should be defined, mainly revolving around accurate imaging.

## Figures and Tables

**Table 1:** Studies included in our review.

Author	Study Cohort	Endoleaks
	Chimney	
Ahmad et al. (2020) [3]	54	8 (14.8%)
Bosiers et al. (2016) [4]	95	10 (10.5%)
Huang et al. (2019) [5]	226	37 (16.3%)
Kanaoka et al. (2018) [6]	55	22 (40%)
Mangialardi et al. (2014) [7]	26	6 (23%)
Voskresensky et al. (2017) [8]	27	2 (7.4%)
Wang et al. (2017) [9]	122	13 (10.6%)
Wang et al. (2017) [10]	23	3 (13%)
Zhao et al. (2019) [11]	234	75 (32%)
Zou et al. (2015) [12]	35	5 (14.2%)
Total	897	181 (20.1%)
	Fenestrated	
Canaud et al. (2019) [14]	35	0
Canaud et al. (2019) [15]	17	0
Chassin-Traubert et al. (2020) [16]	50	1 (2%)
Fernandez-Alonso et al. (2020) [13]	14	3 (21.4%)
Furuta et al. (2020) [1]	205	7 (3.4%)
Iwakoshi et al. (2015) [17]	32	3 (9.3%)
Kurimoto et al. (2015) [18]	37	12 (32.4%)
Tan et al. (2020) [19]	7	0
Tsilimparis et al. (2020) [20]	44	0
Yap et al. (2018) [21]	5	0
Zhou et al. (2017) [22]	42	0
Zhu et al. (2018) [23]	10	0
Zhu et al. (2020) [24]	58	2 (3.4%)
Total	556	28 (5%)
	Branched	
Chen et al. (2013) [34]	122	12 (9.8%)
Clough et al. (2017) [35]	30	0
Czerny et al. (2017) [47]	15	1 (6.6%)
Czerny et al. (2021) [36]	43	2 (4.6%)
Dai et al. (2015) [37]	93	0
Ferrer et al. (2018) [38]	7	0
Ferrer et al. (2019) [39]	24	0
Kawajiri et al. (2018) [40]	11	2 (18.1%)
Li et al. (2021) [41]	16	1 (6.2%)
Patel et al. (2016) [42]	22	4 (18.1%)
Tazaki et al. (2017) [43]	217	9 (4.14%)
Tsilimparis et al. (2017) [44]	20	1 (5%)
Tsilimparis et al. (2018) [45]	54	2 (3.7%)
Verscheure et al. (2019) [46]	70	2 (2.8%)
Total	744	36 (4.8%)
	In situ fenestration	
Hu et al. (2017) [26]	107	0
Katada et al. (2015) [27]	7	0
Kopp et al. (2018) [28]	25	1 (4%)
Le Houreou et al. (2018) [29]	16	0
LiChong et al. (2020) [25]	148	7 (4.7%)
Luo et al. (2020) [30]	50	0
Redlinger et al. (2013) [31]	22	0
Wang et al. (2021) [32]	5	0
Zhao et al. (2020) [33]	130	4 (3%)
Total	510	12 (2.3%)
	Comparative studies	
Konstantinou et al. (2020) [48]	36 (19 Fenestrated, 17 Branched)	1 type (Branched)
XiaoHui et al. (2018) [49]	85 (67 Chimney, 18 In situ fenestration)	1 type 1 (Chimney)

## Data Availability

All data can be found in open-access papers and public records online.
